# The Effect of Ceramide Ratio on the Membrane Curvature of Mimetic Models of Matrix Vesicles

**DOI:** 10.1021/acsphyschemau.5c00010

**Published:** 2025-06-09

**Authors:** Diane C. A. Lima, Guilherme Volpe Bossa, Pietro Ciancaglini, Ana P. Ramos, Thereza A. Soares

**Affiliations:** † Department of Chemistry, 124588 FFCLRP, University of São Paulo, 14040-901 Ribeirão Preto, Brazil; ‡ Institute of Mathematical and Physical Sciences, 28040 Universidad Austral de Chile, 5090000 Valdivia, Chile; § Hylleraas Centre for Quantum Molecular Sciences, University of Oslo, 0315 Oslo, Norway

**Keywords:** ceramide, sphingomyelin, sphingomyelinase, membrane curvature, matrix vesicles, Langmuir−Blodgett, molecular dynamic simulations

## Abstract

The lipid composition of membrane systems plays a critical role in regulating their structural dynamics and curvature, particularly in the biological context of matrix vesicles (MVs) formation during bone mineralization. Recent evidence suggests that the lipid composition of MVs, particularly the balance between sphingomyelin (SM) and ceramide (CER), influences their curvature and stability. We report on the impact of SM and CER ratios on membrane curvature through surface pressure–area isotherm measurements and molecular dynamics (MD) simulations at atomistic and coarse-grained levels. Our findings reveal that increasing the CER content up to 25% significantly enhances membrane curvature, as demonstrated by changes in experimental compressibility moduli and lateral pressure profiles. The lateral pressure profiles and spontaneous bending moments calculated from MD simulations of osteoblast-mimetic membrane models suggest a strong propensity for curvature, particularly in asymmetrical bilayers. It also reveals the role of CER-rich domains in the stabilization of membrane curvature, potentially facilitating the budding processes critical for MVs formation in osteoblasts. These findings underscore the critical role of lipid composition in the mechanisms driving MVs biogenesis.

## Graphical abstract

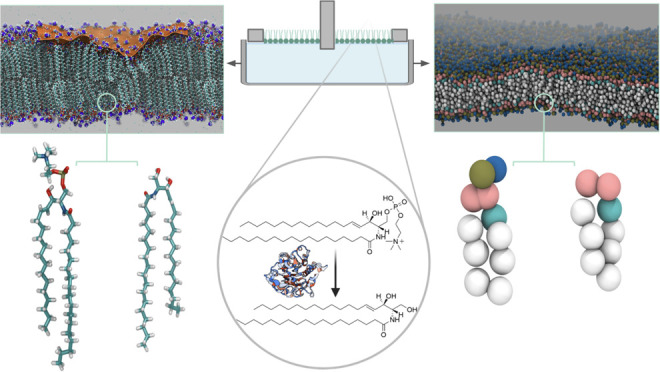

## Introduction

Biomineralization is the process by which minerals are deposited into biological tissues. In vertebrates, crystalline calcium phosphate or hydroxyapatite [Ca_10_(PO_4_)_6_(OH)_2_] is the main mineral in the skeletal system.^1,2^ The nucleation of hydroxyapatite crystals occurs within spherical bodies, known as matrix vesicles (MVs). MVs are small (20–200 nm) extracellular vesicles budding from the cellular membrane of chondrocytes, osteoblasts, and odontoblasts. MVs are enriched in tissue-nonspecific alkaline phosphatase. This enzyme catalyzes the hydrolysis of inorganic pyrophosphate (PPi) to form phosphate ions (PO_4_^3–^), which reacts with Ca^2+^ ions to form hydroxyapatite crystals. As PPi inhibits the formation of hydroxyapatite crystals, alkaline phosphatase plays a crucial role in the biomineralization process. The accumulation of Ca^2+^ and PPi ions in the MVs leads to the progressive growth of the hydroxyapatite crystals, and ultimately, the rupture of the MVs membrane with the release of the material on the collagen scaffold.^3^ For this reason, the secretion of MVs is fundamental for the development and maintenance of skeletal structures, ensuring structural integrity, mechanical support, and cellular regulation.^4^ Disturbances in this delicate balance can lead to mineralization-related disorders, including ectopic mineralization, where minerals form deposits in soft tissues such as blood vessels, cartilage, and tendons.^5^

The bioactive sphingolipid ceramide (CER) has emerged as a key molecule in the biomineralization process (Figure 1d).^1,6^ CER is formed through the hydrolysis of sphingomyelin (SM), a major sphingolipid in the outer leaflet of plasma membranes, by sphingomyelinase (SMase) in a rapid and localized reaction.^5,7^ CER constitutes less than 1 mol % of cell membrane.^8^ The SMase conversion of SM to CER in plasma membranes is thought to promote membrane curvature and vesiculation with the formation of MVs.^6^ Indeed, the inhibition of SMase was shown to decrease the formation of ceramide, prevent MVs release, and lead to reduced biomineralization.^5,9^ Although the pivotal role of CER in MVs biogenesis is well established, the precise molecular mechanism underlying such a role remains poorly understood. This is partially due to experimental difficulties associated with the production, isolation, and characterization of homogeneous vesicle populations.^10^

**Figure 1:**
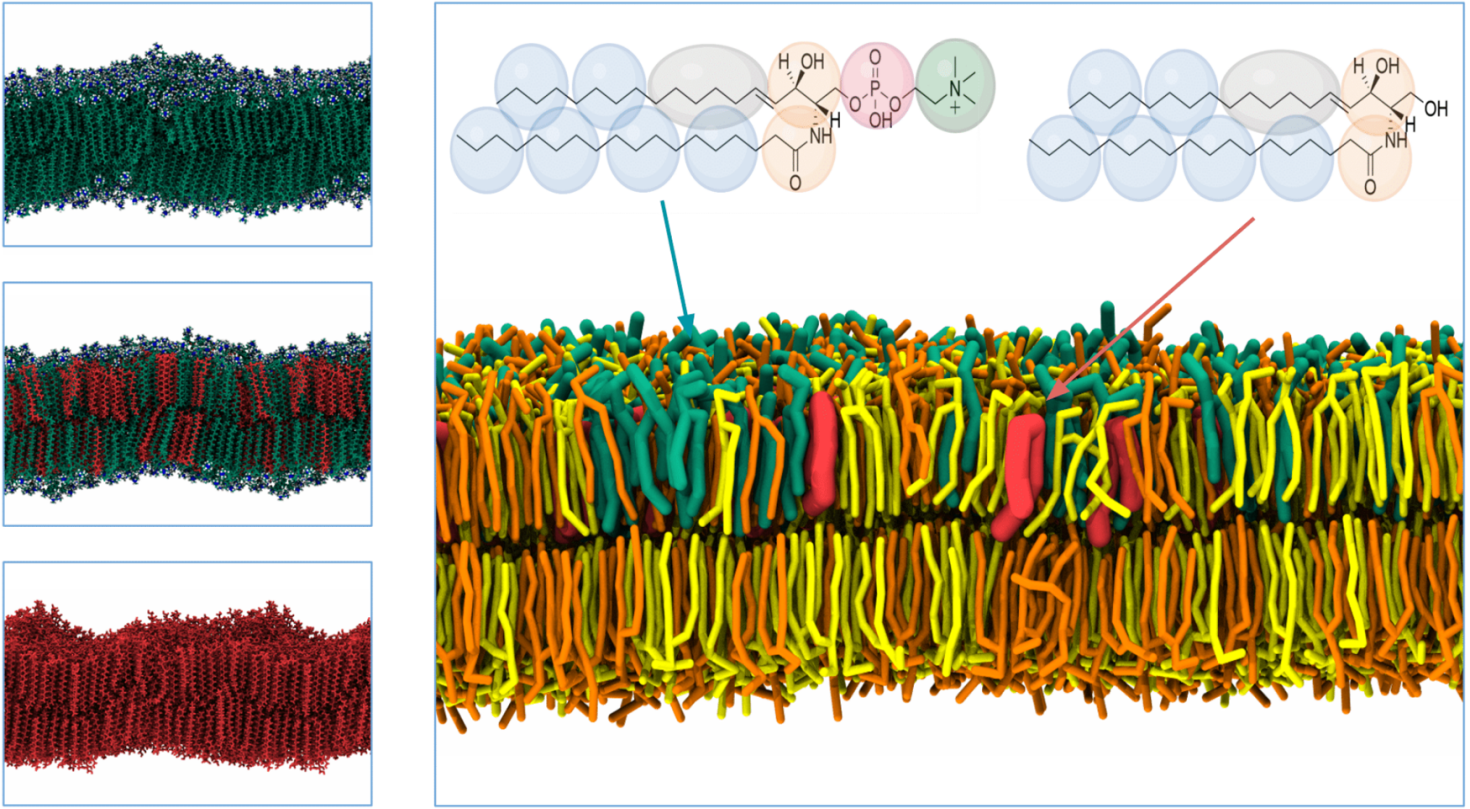
Atomistic representations of bilayers with (a) 100% SM, (b) 50% SM and 50% CER, and (c) 100% CER. SM is shown in green, and CER in red. The nitrogen atom from the choline group of SM is highlighted in blue. (d) Coarse-grained representation of an asymmetrical bilayer mimicking the osteoblast membrane (CG_3_). DPPC and DPPE are depicted in orange and yellow, SM in green, and CER in salmon. Both the molecular structure and coarse-grained beads are illustrated.

On the other hand, the physicochemical behavior of sphingomyelin- and ceramide-rich model membranes has been extensively characterized.^8,11–15^ In fact, the current understanding of the physical and mechanical properties of CER-enriched models attests to the uniqueness of the CER chemical structure. Its small polar headgroup composed of hydroxyl and amide groups can be simultaneously involved in multiple hydrogen bonds. Therefore, CER displays a tight molecular packing and melting transition temperatures above 90 °C.^16^ The addition of CER to SM membranes leads to a significant increase of the viscosity and stiffness of the membrane.^17^ In phospholipid membranes, CER induces the formation of enriched gel or liquid-condensed domains, which are highly ordered and have a low lateral diffusion.^14,18–21^ When mixed with phospholipids, CER coexists as solid, liquid-condensed, and liquid-expanded states over a range of temperatures^22^ and stabilizes highly ordered gel (*L*_β_) over the liquid-crystalline (*L*_α_). Furthermore, CER promotes lamellar-to-hexagonal transitions which can modulate local membrane curvature.^22,23^ Therefore, these and other reports have provided a comprehensive characterization of the CER-rich model membranes. However, the extrapolation of such measurements to cellular processes such as MVs biogenesis remains a nontrivial task. In this work, we have approached the effect of different ratios of CER and SM on the surface curvature of osteoblast membrane models by combining surface pressure–area isotherm measurements and MD simulations at the atomistic (AT) and coarse-grained (CG) resolutions. AT and CG MD simulations offer complementary views of the membrane behavior. Atomistic models capture detailed interactions and fine structural features, while coarse-grained models enable the exploration of larger systems and longer time scales. Together, they bridge molecular precision and mesoscopic dynamics, providing a comprehensive understanding of membrane phenomena. We have also calculated the spontaneous bending moment and Gaussian modulus to characterize the membrane’s intrinsic curvature preferences and its energetic contributions to shape transformations, such as vesicle budding or fusion. We have determined the optimal ceramide content for membrane curvature through sphingomyelinase (SMase) activity and shown that at this ratio of CER/SM composition membranes display the greatest curvature if compared to the different CER/SM ratios investigated through atomistic MD simulations. In our study, we show that asymmetrical MVs membrane models with optimal ceramide content exhibit a spontaneous bending moment and Gaussian modulus approximately twice as high as those in corresponding symmetrical control membranes. These findings attest to the critical role of CER-enriched regions in stabilizing highly curved structures, with important implications for the formation of MVs.

## Methodology

### Experimental Procedure

#### Materials

The lipids *N*-stearoyl-d-erythro-sphingosylphosphorylcholine (SM) and *N*-stearoyl-d-erythro-sphingosine (CER) were purchased from Avanti Polar Lipids (purity 99%). Both the lipids are composed of two carbon chains bearing 18 carbons each and a single double bond in one of the chains, denoted as 18:1/18:0.

#### Langmuir–Blodgett Monolayers Preparation

To prepare the Langmuir–Blodgett monolayers (LB), the lipid solution was prepared at a final concentration of 2 mM by initially solubilizing the lipids in a solvent mixture of 7:3 chloroform and methanol, both of analytical grade, purchased from Merck. Pure and mixed monolayers containing 100% SM; 75 mol % SM/25 mol % CER; 50 mol % SM/50 mol % CER; 25 mol % SM/75 mol % CER; and 100% CER were prepared by spreading the lipid solution at the air–liquid interface of a Langmuir trough (KSV Nima), with the aid of a microsyringe. After 5 min needed for solvent evaporation, the mechanical barriers of the trough were closed at a rate of 10 mm/min, and the surface pressure data as a function of the surface area was acquired by means of a Wilhelmy plate. All of the experiments were carried out in an air-conditioned room (25 ± 1 °C). We note that direct comparisons between molecular dynamics (MD) simulations of bilayer membranes and LB isotherm measurements of lipid monolayers are subject to inherent limitations. For this reason, we aimed to interpret the experimental and computational data as rather complementary. This approach acknowledges that while MD simulations of a bilayer provide a more realistic representation of a minimal model of osteoblast membrane, LB measurements for a monolayer are more feasible and accurate than those with bilayers. LB monolayers have been extensively used to mimic cell membranes because they allow precise control over composition and molecular packing, making them ideal for studying surface interactions.^24–26^ By combining both approaches, we can integrate and complement the detailed molecular-level data provided by experiments and simulations.

#### Excess Area Calculation

We have calculated the excess molecular area (*A*_exc_) for the binary mixtures as it can provide useful insight into the nature of interactions between lipid molecules, lipid packing behavior, and phase transitions.^27–29^ If the observed *A*_exc_ in the mixture deviates from the area predicted by simple additive behavior, this indicates nonideal mixing. Positive *A*_exc_ suggests repulsive interactions, while a negative excess molecular area implies attractive interactions. The excess molecular area *A*_exc_ can be expressed as

1 $$A_{\mathrm{exc}}=A_{\mathrm{mix}}-A_{\mathrm{ideal}}$$

$$A_{\mathrm{exc}}=A_{\mathrm{mix}}-{(A}_{\mathrm{CER}}\cdot X_{\mathrm{CER}}+A_{\mathrm{SM}}\cdot X_{\mathrm{SM}})$$

where *A*_exc_ represents the excess molecular area value from experiment or simulation of a mixture, *A*_CER_ stands for the molecular area of pure CER, *A*_SM_ denotes the molecular area of pure SM, *X* indicates the mole fraction, where *X*_CER_ denotes the mole fraction of CER, and *X*_SM_ represents the mole fraction of SM. The *A*_exc_ corresponds to repulsive interactions if *A*_mix_ > *A*_ideal_, attractive interactions if *A*_mix_ < *A*_ideal_, or ideal behavior if *A*_mix_ = *A*_ideal_.^29^

#### Excess Gibbs Energy

The excess Gibbs energy (Δ*G*_exc_) was calculated using a protocol adapted for experimental Langmuir data.^29,30^ It takes into account the *A*_exc_ values, and it is given by

2 $$\Delta G_{\mathrm{exc}}=N_{A}\int_{\pi }^{0}A_{\mathrm{exc}}d\pi^{\prime }$$

where *N*_A_ is Avogadro’s constant, *A*_exc_ is the excess molecular area and the function of the surface pressure, and *d*π′ represents an infinitesimally small change in the surface pressure π.

#### Compressibility Modulus

The physical states (gaseous, liquid-expanded, liquid-condensed, and solid) and phase transitions in Langmuir monolayers can be classified in relation to the compressibility modulus (*C*_s_^–1^). This parameter considers how the lipid monolayers respond to pressure changes when the surface area is changed. *C*_s_^–1^ can also be related to the fluidity of the monolayer.^24,31,32^ It is defined as follows

3 $${C_{s}}^{-1}=-A_{\pi }{\left(\frac{d\pi }{dA}\right)}_{T}$$

where *A*_π_ is the area per molecule at the indicated surface pressure π and ${\left(\frac{d\pi }{dA}\right)}_{T}$ is the partial derivative of π vs A isotherm.

#### Sphingomyelinase (SMase) Enzymatic Assays

The activity of SMase on a monolayer composed of SM was calculated using Langmuir monolayers.^14^ For this, a chloroformic SM solution was spread at the air–liquid interface of the Langmuir trough, previously filled with 5 μL of SMase added to 140 mL of an aqueous-buffered subphase, composed of 50 mM Tris-HCl (pH 7.4) containing 2 mM MgCl_2_. Upon compression, the resultant profile of the π × *A* isotherm obtained in the presence and absence of SMase was used to analyze the changes induced by the enzymatic conversion process. For the enzyme assays, SMase obtained from Merck was used. The enzyme was supplied in an aqueous solution with activity ranging from 100 to 300 units/mg. In biological context, SMase catalyzes the conversion of SM into CER.

### Computational Procedure

#### Atomistic Simulations

Molecular dynamics (MD) simulations with atomistic models (AT) were carried out for membranes composed of different proportions of sphingomyelin (18:1/18:0) (SM) and ceramide (18:1/18:0) (CER) lipids; see Figure 1 and Table 1. Each membrane was built with 256 lipids using the CHARMM-GUI Web site.^33,34^ The CHARMM36 force field was used with the TIP3 water model and 150 mM of CaCl_2_.^34,35^ Duplicates of distinct molecular systems were simulated for 1 μs each. Simulated systems are presented in Table 1. Energy minimization was performed for all the systems using the Steepest Descent algorithm until a mean force of less than 1000 kJ mol^–1^ nm^–1^ was achieved. The equilibration phase was initially performed in the NVT ensemble for 250 ps with a time step of 0.001 ps. Subsequently, the equilibration was continued in the NPT ensemble for 125 ps and a time step of 0.001 ps, followed by 300 ns with a time step of 0.002 ps. In the equilibration phase, temperature control was performed via the Berendsen thermostat^36^ with a reference value of 303.15 K and coupling constant of 1 ps. The Berendsen barostat was used to keep the pressure constant around 1 atm with a pressure coupling of 5.0 ps.^37^ The semi-isotropic coordinate scaling was applied with an isothermal compressibility of 4.5 × 10^–5^ (kJ mol^–1^ nm^–3^)^−1^. The production phase was carried out in the NPT ensemble with a time step of 0,002 ps. The temperatures of solute and solvent were controlled by separately coupling them to the Nose-Hoover thermostat with relaxation time of 1.0 ps and temperature of 303.15 K.^38^ The pressure was maintained at 1 atm by using the Parrinello–Rahman barostat with a 5.0 ps pressure coupling constant, semi-isotropic coordinate scaling and isothermal compressibility of 4.5 × 10^–5^ (kJ mol^–1^ nm^–3^)^−1^.^39^ The geometry of the water molecules and the length of the bonds between the atoms composing the solute were constrained using the LINCS algorithm.^37^ Nonbonded interactions were treated with a cutoff of 1.2 nm. Long-range electrostatic interactions were treated by applying the Particle Mesh Ewald approximation, projecting charges onto a 0.12 nm grid with cubic interpolation for the reciprocal space calculations.^40,41^ MD simulations were performed with the GROMACS software v. 2021.3 ^42^ and trajectories were written at 1000-step intervals. The same procedure was applied at 310.3 and 333.3 K to assess potential temperature-dependent trends in the simulated properties of the lipid mixtures. The SuAVE software was used for analyses of curvature-dependent membrane properties, e.g., area per lipid (*A*_L_), curvature order parameter (*S*_C_), and membrane thickness (*D*_HH_), see Table SI-1.^43,44^

**Table 1:** Atomistic (AT) and Coarse-Grained (CG) Simulations^a^

	number of molecules							
systems	PC	PE	SM	CER	water	ions	lipid percentages	time (μs)
AT_100‑SM_			512		18,960	48 Ca^2+^, 96 Cl^–^	100% SM	1
AT_75‑SM_			384	128	19,482	50 Ca^2+^, 100 Cl^–^	75% SM, 25% CER	1
AT_50‑SM_			256	256	20,020	51 Ca^2+^, 102 Cl^–^	50% SM, 50% CER	1
AT_25‑SM_			128	384	20,585	54 Ca^2+^, 108 Cl^–^	25% SM, 75% CER	1
AT_100‑CER_				512	21,211	56 Ca^2+^, 112 Cl^–^	100% CER	1
CG_1_	2000	2000			42,764	514 Na^+^, 514 Cl^–^	100% (PC + PE)	12
CG_2_			3000	1000	42,007	567 Na^+^, 567 Cl^–^	75% SM, 25% CER	12
CG_3_	1700	1700	450	150	43,977	572 Na^+^, 572 Cl^–^	*L*_Up_: 70% (PC + PE), 30% (75% SM + 35% CER)	12
							*L*_Lo_: 100% (PC + PE)	
CG_vesicle_	1643	1645	643	205	215,972	526 Na^+^, 526 Cl^–^	*L*_Up_: 70% (PC + PE), 30% (75% SM + 35% CER)	10
							*L*_Lo_: 100% (PC + PE)	
							*L*_vesicle_: 75% SM, 35% CER	

a The AT simulations have the same lipid proportion in both monolayers. For clarity in asymmetrical systems, *L*_Up_ and *L*_Lo_ refer to the upper and lower leaflets, respectively.

#### Coarse-Grain Simulations

Atomistic (AT) and coarse-grained (CG) MD simulations have been shown to be complementary approaches in the fields of computational chemistry and physics. While AT simulations provide finer chemical details, albeit at a great computational cost, CG models enable the simulation of larger system sizes and longer time scales that are not feasible with atomistic models due to computational limitations. For this reason, coarse-grained (CG) MD simulations were performed for membranes composed of DPPC, DPPE, CER, and SM, as described in Table 1. The choice of lipid composition reflects that of a biological membrane in osteoblast cells, although the proportions of CER and SM in these cells are not precisely known. For this reason, the ratio of CER and SM was estimated from the SMase enzyme assay (see the Experimental Procedure section), which shows that enzymatic activity ceases once ca. 25% of SM was converted to CER. Consistently, this is also the SM/CER ratio yielding most curvature among the atomistic simulations with different ratios of the two lipids. Furthermore, a similar SM/CER ratio was previously shown to promote change of CER-rich domains from circular to elongated shape in giant unilamellar vesicles.^21,45^ The CHARMM-GUI Web site^34,46^ was used to build the atomic coordinates for the simulated systems combined with the MARTINI 2 force field.^46–48^ The system was solvated using the MARTINI CG water model and 150 mM of NaCl_2_. The systems were geometry optimized for 10,000 steps using the Steepest Descent algorithm and equilibrated in the NPT ensemble with the progressive increase of the time step from 0.002 to 0.020 ps in increments of 0.005, 0.010, and 0.015 ps. Each incremental time step was conducted for 1 ns, and the system was equilibrated for 200 ns. During the equilibration, the Berendsen barostat^36^ was used with a semi-isotropic pressure coupling of 5.0 ps, whereas in the production phase, the Parrinello–Rahman barostat^39,49^ was applied with a pressure coupling of 12.0 ps. Throughout the simulation, the reference pressure was kept at 1.0 bar and the compressibility at 3 × 10^–4^ (kJ mol^–1^ nm^–3^)^−1^. The v-rescale algorithm was employed for temperature control with a coupling time constant of 1.0 ps at 303.15 K.^46^ The Verlet buffer was used with a tolerance of 0.005. Long-range interactions were treated with a cutoff scheme after 1.1 nm.^50^ The PME approximation was used to treat long-range electrostatic interactions, and van der Waals interactions were accounted for using the vdW-modifier set to Potential-shift-Verlet.

#### Calculation of the Elastic Constants

The lateral pressure profile (LPP) was determined using the following equation

$$\mathrm{LPP}\left(z\right)=\frac{\left[P_{\mathrm{xx}}\left(z\right)+P_{\mathrm{yy}}\left(z\right)\right]}{2}-P_{\mathrm{zz}}\left(z\right)$$

where *P*_*xx*_, *P*_*yy*_, and *P*_*zz*_ are the components of the stress tensor extracted from the MD simulations, and *z* is the Cartesian coordinates defined as the axis normal to the membrane surface. The lateral pressure profiles are presented as a function of coordinate *z* for the three CG membrane simulations (Figure 7 and Table 1). Given that the membrane thickness is measured from a position *z = d*_–_ to *z = d*_+_, the product of the bending stiffness *K*_C_ by the spontaneous curvature *c*_0_ can be calculated directly from the lateral pressure profile above via

$$K_{C}c_{0}=\int_{d_{-}}^{d_{+}}z\mathrm{LPP}\left(z\right)dz$$

Throughout this work, we refer to the product *K*_C_ *c*_0_ as the spontaneous bending moment.^51^ The knowledge of the lateral pressure profile also allows us to calculate the Gaussian modulus (*K̅*_G_) using the following equation

$${\mathrm{K̲}}_{G}=-\int_{d_{-}}^{d_{+}}z^{2}\mathrm{LPP}\left(z\right)dz$$

where we have assumed the membrane midplane lies at *z* = 0. The values of spontaneous bending moment and of the Gaussian modulus are displayed in Table 3 for each membrane composition.

## Results and Discussion

We compared the molecular area (*A*_L_) values obtained from the MD simulations and LB trough assays for different ratios of SM and CER (Table 2). *A*_L_ values have also been reported in the literature for single lipid membranes composed of C18 SM and C18 CER. These *A*_L_ values range from 47 to 55 Å^2^ for pure SM, and from 40 to 45 Å^2^ for pure CER.^52–56^ Therefore, the computational and experimental *A*_L_ values are consistent with previous measurements in the literature for single lipid membranes (Table 2). *A*_L_ values for binary mixtures of C18 SM and CER have not been reported in the literature. Although *A*_L_ values for binary mixtures of C16 SM and C16 CER have been previously reported,^14,57^ the dependence of the *A*_L_ and compressibility modulus (Cs^1–^) on the length of the saturated acyl chain hamper a quantitative comparison between binary mixtures containing C18 versus C16 acyl chain lengths.

**Table 2:** Comparison of Molecular Area and Compressibility Modulus at Different Surface Tensions from Langmuir–Blodgett Values through Experiments and Atomistic MD Simulations

		experiment			simulations			
	composition	30 mN/m	25 mN/m	20 mN/m	303 K	310 K	333 K	literature
*A*_L_ (Å^2^)	SM	50	54	62	54.1			47–61 ^52–56^
	25-CER	39	44	49	49.2	49	50	
	50-CER	37	40	45	45.9	47	47	
	75-CER	39	41	44	42.8	43	44	
	CER	38	41	43	45.1			40–49 ^52–56^
	SMase_conver_	42	46	50				
Cs^1–^ (mN/m)	SM	52	46	46				50 ^29^
	25-CER	49	43	42				
	50-CER	61	55	53				
	75-CER	81	73	66				
	CER	110	100	81				

Nonetheless, it is possible to compare trends for the two groups of binary mixtures, as long as the temperatures are within the same range. In this context, the behavior of the binary mixtures of C18 SM and CER is consistent with the trend reported in the literature,^14^ which shows a decrease of *A*_L_ as the ratio of CER increases. It is noteworthy that for both chain length binary mixtures, *A*_L_ values become smaller than expected as the ratio CER/SM increases (Figures 2a and SI-1).^14^

**Figure 2:**
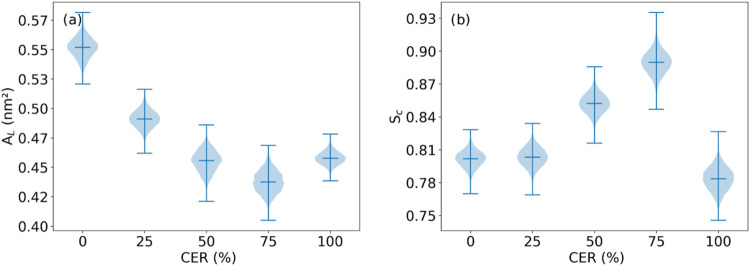
(a) Average area per lipid *A*_L_ and (b) average membrane curvature *S*_C_ for binary mixtures of CER and SM, calculated for AT-simulated systems. The violin plots represent the data set distribution where the width of the violin indicates the data density at different values. The central line represents the median, and the box shows the interquartile range. The overall shape of the plot reveals the spread and skewness of the distribution. The averages were calculated over the final 100 ns of the simulations.

We also examined the excess molecular area *A*_exc_ and excess Gibbs energy (Δ*G*_exc_) for pure and binary lipid systems (Figure 3). The *A*_exc_ describes the difference between the observed molecular area and those predicted from ideal mixing (eq 1). In this context, the decrease of *A*_L_ with the increase of CER amount is driven by a favorable interaction (*A*_mix_ < *A*_ideal_) between SM and CER (Figure 3a), in which SM-CER interactions are more thermodynamically favorable than CER-CER and SM-SM interactions. This is consistent with the strong attraction between SM and CER previously reported by other groups.^14,58^ For instance, at 25 mN/m, the 50% CER system exhibits an average *A*_L_ value of 40 Å^2^, whereas for an ideal equivalent lipid mixture (*A*_mix_ = *A*_ideal_), the expected *A*_L_ value is 47.5 Å^2^.^27^ The strong attractive interaction between SM and CER is further supported by the negative values of the excess Gibbs energy (Δ*G*_exc_) for the binary systems (Figure 3b).^29^ These negative values are associated with favorable interactions between lipids in the mixed systems.

**Figure 3:**
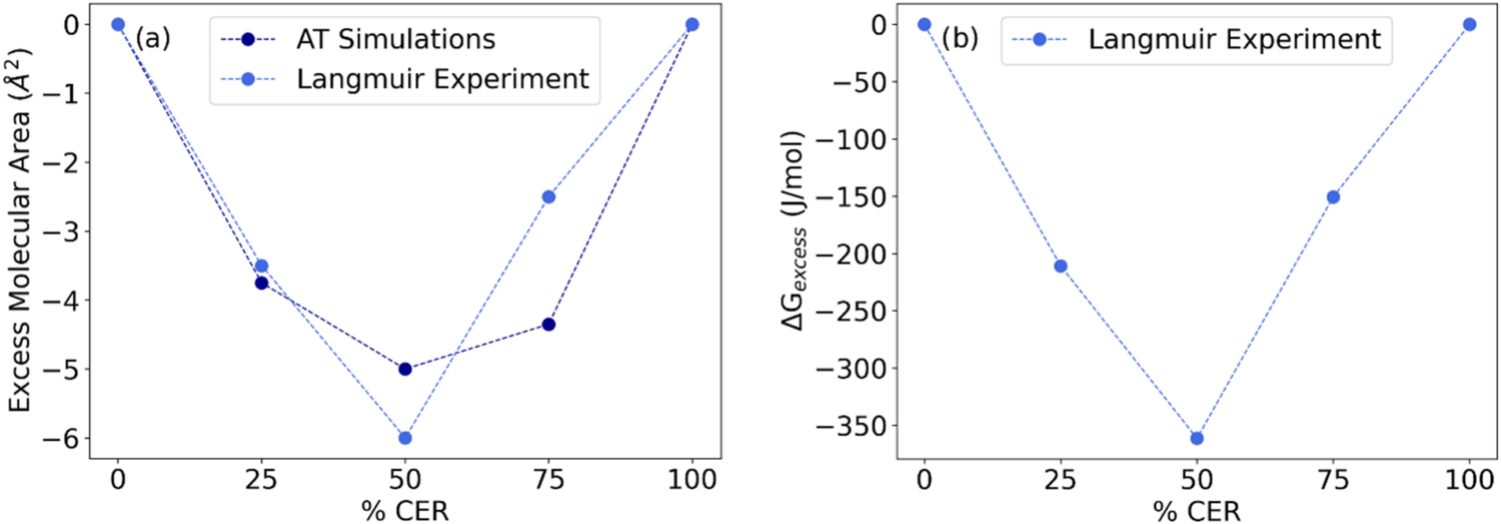
(a) Excess molecular area (*A*_exc_) obtained from experimental LB films (blue) and AT simulations at 303 K (dark blue) and (b) the excess Gibbs energy (Δ*G*_exc_) calculated from experimental LB films for pure or binary mixtures of CER and SM.

The system with equal amounts of the CER and SM has the most favorable Δ*G*_exc_, whereas the two remaining binary systems show less favorable Δ*G*_exc_ values (Figure 3b). These findings point to a higher energetic cost to deform the 50% CER membrane compared to its counterparts, namely, 25% CER and 75% CER. This assumption is consistent with previous experimental reports demonstrating that C16 SM and C16 CER mixtures cannot form vesicles when CER exceeds 40% of the mixture composition.^14^ Altogether, it can be argued that forming vesicles from a membrane with a 50% CER ratio may have a higher energy cost, making it thermodynamically unfavorable. From this perspective, the 25% CER 75% SM system emerges as the most likely composition to exhibit membrane deformation from its spontaneous shape.

Subsequently, we measured the compressibility modulus (Cs^1–^) for different ratios of SM and CER via LB trough assays (Table 2 and Figure SI-1). The Cs^1–^ provides information about the elasticity of the monolayer, indicating how much pressure is required to change the area per molecule in the monolayer. Hence, higher values of Cs^1–^ indicate that the monolayer is more resistant to compression due to a more rigid or densely packed structure.^24^ The measured Cs^1–^ value for pure SM is consistent, qualitatively and quantitatively, with the available literature.^59^ The measured Cs^1–^ increases with the concentration of CER, once again, with the exception of the 25% CER system (Table 2). CER has been extensively reported to raise the Cs^1–^ value of SM membranes due to the CER-induced condensation effect.^17,57^ The 25% CER is an exception to the trend with Cs^1–^ values slightly lower than pure SM (Table 2), indicative of the fact that the former can be compressed with less resistance than the remaining binary mixtures. Remarkably, the same ratio of CER to SM emerged from the LB trough assay upon the conversion of SM to CER by sphingomyelinase (SMase; Figure 4). The resulting isotherm profile from the SMase conversion assay is strikingly analogous to the isotherm measured for the 25% CER system (Figure 4). This is so despite expected differences in the surface topography of SM/CER systems resulting from the SMase-driven reaction and that of premixed SM/CER systems of the same lipid composition.^60,61^

**Figure 4:**
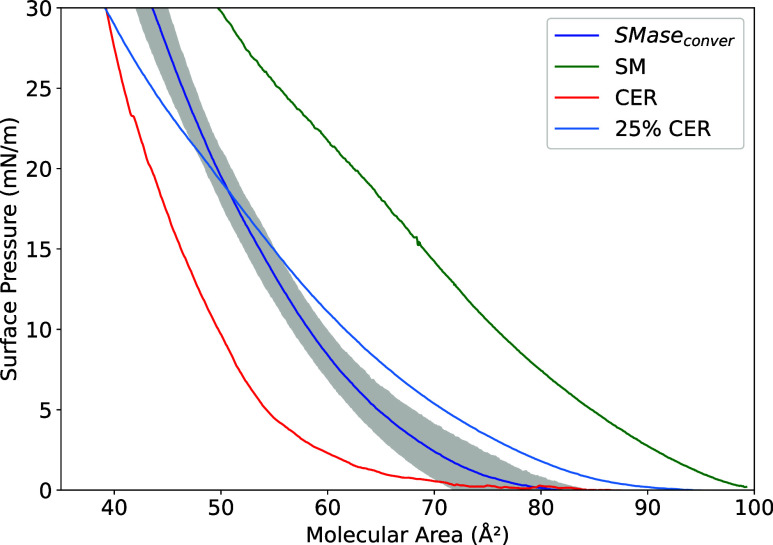
Experimental surface pressure isotherms versus molecular area for pure and SMase-converted binary mixtures of CER and SM. Isotherms for pure SM (green), pure CER (red), and the SM-CER ratio resulting from the SMase catalytic conversion of SM into CER (dark blue). The control isotherm (light blue) corresponds to a composition of 75% SM and 25% CER.

The theory of elasticity defines the relation between the surface compressibility modulus (Cs^1–^ or *K*_A_) and the bending modulus (*K*_C_) for thin films or membranes of uniform thickness. It establishes that higher Cs^1–^ values correspond to higher *K*_C_ values, indicating that the material is more resistant to bending deformation. Accordingly, we have examined the surface curvature of the AT simulations to compare with the LB trough measurements of Cs^1–^ (Figure 2b and Table 2). The curvature order parameter (*S*_C_) extracted from the membrane simulation provides an estimate of the average membrane curvature. It describes the distribution of angles (θ) between the *z*-axis and the normal vector of a surface grid on a bilayer. A value of 1 indicates a planar bilayer, while deviations from it indicate an increase in surface curvature.^43^ It can be seen that the 25% CER membrane exhibited a higher deviation from 1 when compared with the other lipid mixtures (Figure 2b). As the CER ratio increased above the 25% threshold, the surface of the membranes became flatter. The trend further persisted for simulations at higher temperatures of 333 K (Figure SI-2). Therefore, the 25% CER system exhibited the most pronounced curvature (Figure 2b) and the lowest Cs^1–^ value (Table 2) among the binary mixtures. However, it should be noticed that the simulated AT membranes are symmetrical, and hence curvature effects in one leaflet tend to be minimized or neutralized by the opposed leaflet of similar composition.

The low compressibility modulus of the 25% CER membrane can be rationalized by considering several factors. First, this composition displays negative excess area and Gibbs free energy values, indicating strong attractive interactions and favorable nonideal mixing between SM and CER. Such interactions likely prevent the formation of rigid, phase-separated domains and result in a more homogeneous and fluid lipid environment. Second, this system exhibits the highest membrane curvature among all tested compositions in atomistic simulations, suggesting that part of the lateral stress may be relieved through membrane deformation (see the discussion hereafter and Figure 6). Third, the similarity between the SMase-generated isotherm and that of the 25% CER system suggests that enzymatic conversion leads to increased disorder or defects in lipid packing, which may also enhance compressibility. Together, these factors explain why this particular composition deviates from the otherwise monotonic increase in compressibility observed with increasing CER content.

In order to examine the effect of membrane asymmetry, we have performed coarse-grained (CG) simulations of a membrane closely mimicking the lipid composition of the membrane vesicle (MV) in osteoblasts (Table 1). This MV membrane model (CG_3_) was composed of DPPC and DPPE (1:1) in the inner leaflet, and the outer leaflet was made of 70% DPPC and DPPE (1:1) and 30% SM and CER (3:1), respectively. The choice of a 25% CER 75% SM ratio was based on our results for the excess molecular area (*A*_exc_) (Figure 3a), excess Gibbs energy (Δ*G*_exc_) (Figure 3b), surface compressibility modulus (Cs^1–^) (Table 2), curvature order parameter (*S*_C_) (Figure 2b), and our current observation that, at equilibrium, SMase converts ca. 25% of SM into CER (Figure 4). We have also performed CG simulations of symmetrical membranes as negative controls. The systems CG_1_ and CG_2_ were composed of binary mixtures of DPPC/DPPE (1:1) and SM/CER (3:1), respectively (Table 1). The average *A*_L_ calculated for these CG simulations reproduced the experimental trend with smaller values for the CER/SM membrane compared to those for DPPC/DPPE (Figure 5a). The calculated average curvature (*S*_C_) for the CG simulations indicates that the MV membrane model (CG_3_) and DPPC/DPPE membrane (CG_1_) remained mostly flat throughout the 12 μs of simulation, albeit some negligible change in surface curvature was observed for the CER/SM membrane (CG_2_) (Figure 5b). An explanation for these observations is that membrane curvature often involves significant reorganization of lipids, which may not be properly sampled through MD simulations due to the high energy barriers associated with such processes.

**Figure 5:**
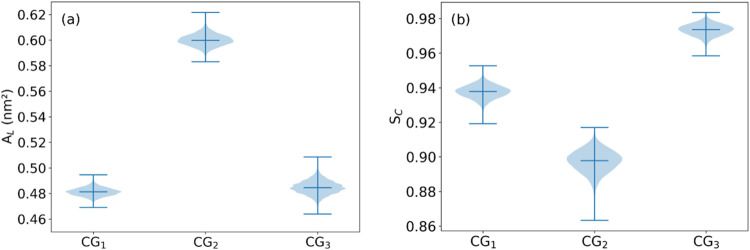
(a) Area per lipid *A*_L_ and (b) average curvature *S*_C_ for CG simulations of the symmetrical membrane consisting of DPPC and DPPE (CG_1_), the symmetrical membrane composed of SM and CER (CG_2_), and the asymmetrical membrane composed of DPPC and DPPE in the inner leaflet and DPPC, DPPE, SM, and CER in the outer leaflet (CG_3_). See Table 1 for details on each system composition.

On the other hand, asymmetrical membranes have been shown to generate curvature to adjust potential mismatches in mechanical and chemical properties between their two leaflets. One way to assess the mechanical properties in membranes is through the calculation of the lateral pressure profile (Figure 6). The lateral stress profile describes how the internal pressure varies within the membrane, underlying important properties, including the spontaneous bending moment (*k*_c_ *c*_0_).^62–65^ The calculated lateral pressure profiles for the simulated systems exhibit the three regimes commonly observed for phospholipid bilayers (Figure 6).^65,66^ There is a repulsive contribution arising mostly from the electrostatic and steric interactions of lipid headgroups, an attractive contribution associated with the lipid–water interfacial tension, and a repulsive contribution due to steric interactions between lipid acyl chains. For the symmetrical CG_1_ membrane, the repulsive contribution at the headgroups is nearly null due to the very small size of the CER headgroups, the large orientational freedom of the SM headgroup and well-characterized ability of all sphingolipids to form extensive intermolecular hydrogen bonding networks.^67^ This is also consistent with the strong attraction between SM and CER.^14,58^ There are two slightly positive and negative peaks corresponding to the lipid–water interfacial tension and the hydrophobic region of the bilayer. The symmetrical CG_2_ follows the same canonical pattern already discussed for atomistic simulations of PC–PE systems.^68^ We can now compare the lateral pressure profile obtained for the asymmetrical MV membrane model CG_3_ with that of the symmetrical control systems (Figure 6).

**Figure 6:**
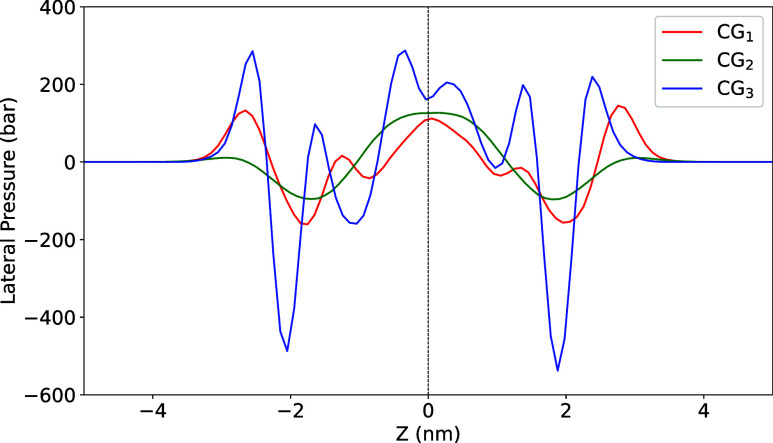
Lateral pressure profile for simulated membranes CG_1_, CG_2_, and CG_3_. The bilayer midplane is positioned at 0 nm. The negative region corresponds to the inner leaflet, while the positive region represents the outer leaflet. The symmetrical CG_1_ system (red) is composed of DPPC and DPPE; the symmetrical CG_2_ system (green) is composed of SM and CER; and the asymmetrical CG_3_ system (blue) is composed of DPPC and DPPE in the inner leaflet, DPPC, DPPE, SM, and CER in the outer leaflet. See Table 1 for the lipid composition of the system.

From the membrane surface inward, the asymmetrical CG_3_ displays a positive peak attributed to the headgroup repulsion, a large negative peak attributed to the lipid–water interfacial tension and positive peaks closer to the center of the bilayer from steric interactions between hydrophobic chains (Figure 6). The small shift in the *z*-position of the corresponding peaks in the two systems can be assigned to differences in membrane thickness (Figure SI-4). Although the lateral pressure profiles of CG_3_ and CG_2_ are qualitatively similar, CG_3_ exhibits significantly greater lateral pressure values (Figure 6). Indeed, the lateral pressure at the headgroup interface is 2 to 3-fold greater for the MV membrane model CG_3_ than for the symmetrical membranes CG_1_ and CG_2_ (Figure 6). As the asymmetry in the lateral pressure profile across the membrane creates a spontaneous bending moment, we have also calculated this quantity for the three systems (Table 3).^62^

**Table 3:** Spontaneous Bending Moment and Gaussian Modulus for Each Composition of the Membrane, Calculated for CG-Simulated Systems

systems	*K*_*C*_ *c*_0_ [pN]	*K̅*_G_ [κ_B_ *T*]
CG_1_	24	–12
CG_2_	59	–52
CG_3_	115	–105

The spontaneous bending moments are 24, 59, and 115 pN for CG_1_, CG_2_, and CG_3_, respectively. Experimental estimates of k_C_ for CER/SM domains in ternary mixtures with POPC are ca. 50 *k*_B_ *T* compared to 7.8 *k*_B_ *T* and 28.9 *k*_B_ *T* for POPC and POPC/SM domains, respectively.^69^ Likewise, experimental estimates of *C*_0_ for binary mixtures of CER and C24 SM are ca. −0.015 nm^–1^, whose magnitude corresponds to curvature with a radius of approximately 66.67 nm as given by *R* = 1/|*C*_0_|.^70^ Therefore, the spontaneous bending moment of 115 pN and the negative value of the corresponding Gaussian modulus calculated for the MV membrane model CG_3_ indicate that it is prone to form highly curved structures, such as small buds or vesicles, and would do so spontaneously under normal conditions.

Lastly, Holopainen and co-workers showed that vesicle budding and shedding can be triggered by the asymmetrical SMase-catalyzed generation of ceramide in giant phosphatidylcholine/sphingomyelin liposomes.^71^ This process was associated with phase separation of CER-enriched domains, emergence of negative spontaneous curvature, and increased *k*_C_ values for CER-containing domains.^71^ Based on this report, we performed an additional CG simulation of the MV membrane model containing a bud composed exclusively of CER/SM (1:3) (CG_4_) (Figure 7). The composition of the bud reflects previous experimental reports, which show the formation of CER/SM domains antecedes alterations of membrane curvature.^14^

**Figure 7:**
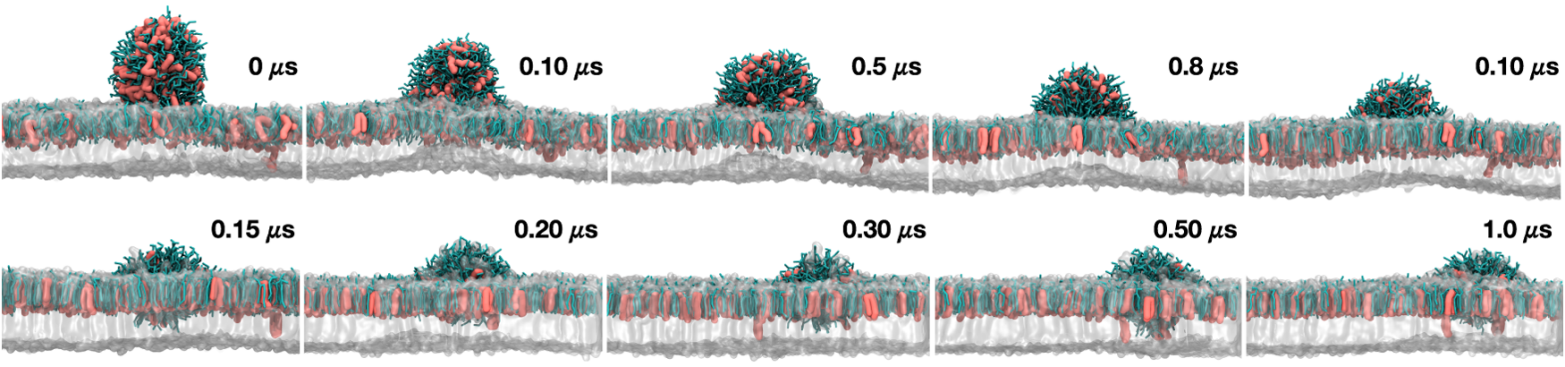
Structure of the bilayer and semivesicle system through simulation. For clarity, DPPC and DPPE are shown in white, while CER is illustrated in salmon, and SM in green.

This setup was aimed to simulate the reverse process of curvature formation without the use of external forces to induce curvature (i.e., tension pressure, osmotic imbalance), which could mask the real contribution of the lipid composition. The goal was to investigate budding stability versus spontaneous CER depletion. It can be seen that the CER/SM bud steadily merges with the outer leaflet of the MV membrane, while it induces a mild negative curvature in the inner leaflet composed exclusively of DPPC/DPPE (Figure 7). As the process evolves to nearly full completion, CER diffuses from the bud into the outer leaflet of the membrane, while the bud merges further with the outer leaflet. This simulation supports the role of ceramide in maintaining the integrity of the bud structure, most likely by stabilizing regions of the increased curvature in the MV membrane. Collectively, our experimental and computational data highlight the important role of the CER in matrix vesicle (MV) biogenesis, corroborating previous experimental findings.

## Conclusions

We investigated the potential role of ceramide (CER) in the modulation of curvature in osteoblast membrane models by combining Langmuir monolayer experiments and molecular dynamics (MD) simulations at both atomistic and coarse-grained resolutions. We have demonstrated that the sphingomyelinase (SMase) activity is optimal at a CER ratio of 25% for the membrane composition used in this report, which also corresponds to the membrane ratio exhibiting the largest curvature and fluidity from atomistic MD simulations. At this specific ratio, the membrane exhibits lower compressibility moduli and increased curvature order parameters compared to those of other CER/SM ratios. We have further shown that the nonideal mixing behavior between sphingomyelin (SM) and CER results in negative excess Gibbs energy, favoring the formation of condensed membrane domains and leading to enhanced membrane packing and stability. MD simulations reveal that the asymmetric distribution of lipids, especially ceramide, in osteoblast membrane models induces significant curvature with spontaneous bending moment and Gaussian modulus values that are twice as large as those observed for symmetrical membranes used as controls. Hence, the osteoblast-mimetic membranes experience significant stress and a strong tendency for curvature under strain. MD simulations also indicate that CER-enriched regions stabilize highly curved structures, which therefore may play **a critical role** in maintaining the integrity of curved membrane regions during vesicle budding and release. Our findings indicate that the enzymatic conversion of SM to CER is crucial for modulating the curvature and budding of matrix vesicles, with CER-enriched regions stabilizing highly curved structures. These findings provide insights into the effect of lipid composition on the membrane curvature and stability in osteoblast membranes, with potential implications for understanding matrix vesicle formation in bone mineralization.

## Supplementary Material



## Data Availability

Input files and scripts are available in the BioMat GitHub. https://github.com/BioMat-USP-RP/Input-files-for-SM-CER-simulations-of-mimetic-models-of-matrix-vesicles

## Notes

Additional experimental isotherms and compressibility modulus; computational analyses for AT systems at 333 K and CG systems; and computational variable descriptions (PDF)
