# Supplementary material for: The Effect of Ceramide Ratio on the Membrane Curvature of Mimetic Models of Matrix Vesicles
Source: ACS Phys Chem Au. 2025 Jun 9;5(5):456–66. doi: 10.1021/acsphyschemau.5c00010 (PMC12464780; doi:10.1021/acsphyschemau.5c00010)
Supplement: Supplementary file 1 [file pg5c00010_si_001.pdf]

The Effect of Ceramide Ratio on Membrane Curvature of Mimetic Models of Matrix Vesicles

Diane C. A. Lima[1], Guilherme Volpe Bossa[2], Pietro Ciancaglini[1], Ana P. Ramos[1], Thereza A. Soares[1,3,*]

[1]Department of Chemistry, FFCLRP, University of São Paulo, 14040-901, Ribeirão Preto, Brazil

[2]Institute of Mathematical and Physical Sciences, Universidad Austral de Chile, 5090000, Valdivia, Chile

[3]Hylleraas Centre for Quantum Molecular Sciences, University of Oslo, 0315, Oslo, Norway

Corresponding author: *thereza.soares@usp.br*

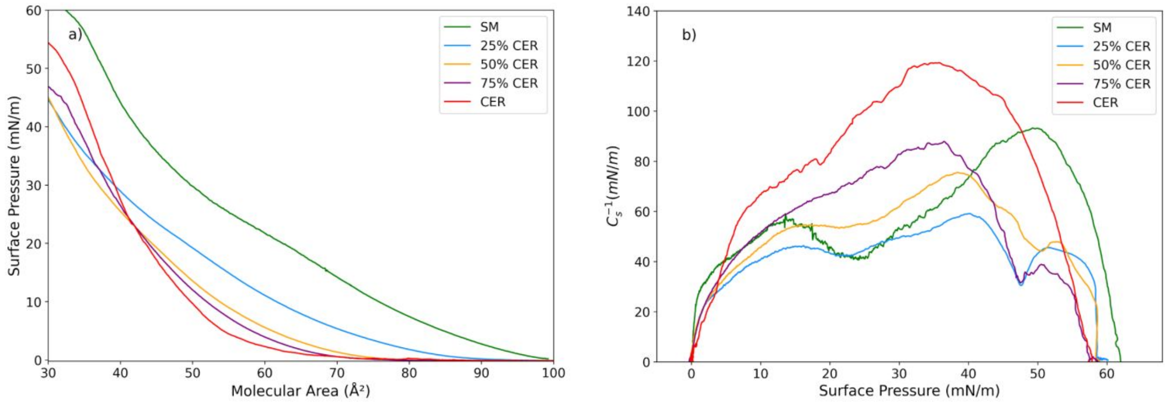

Figure SI-1: a) Experimental isotherms and b) compressibility modulus for monolayers at different lipid ratios of sphingomyelin (SM) and ceramide (CER).

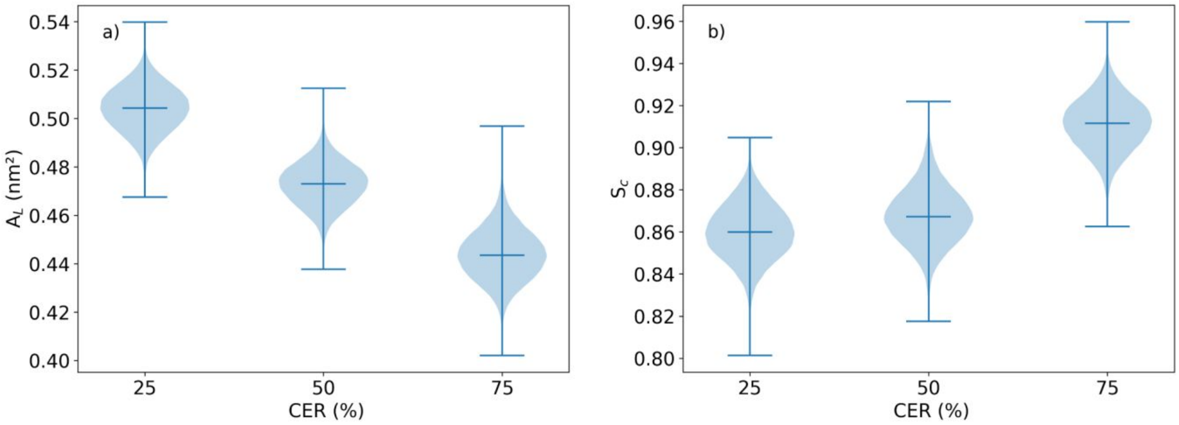

Figure SI-2: Average area per lipid (A_L) and surface curvature (S_c) as a function of the increase in ceramide (CER) for AT simulated systems at 333K.

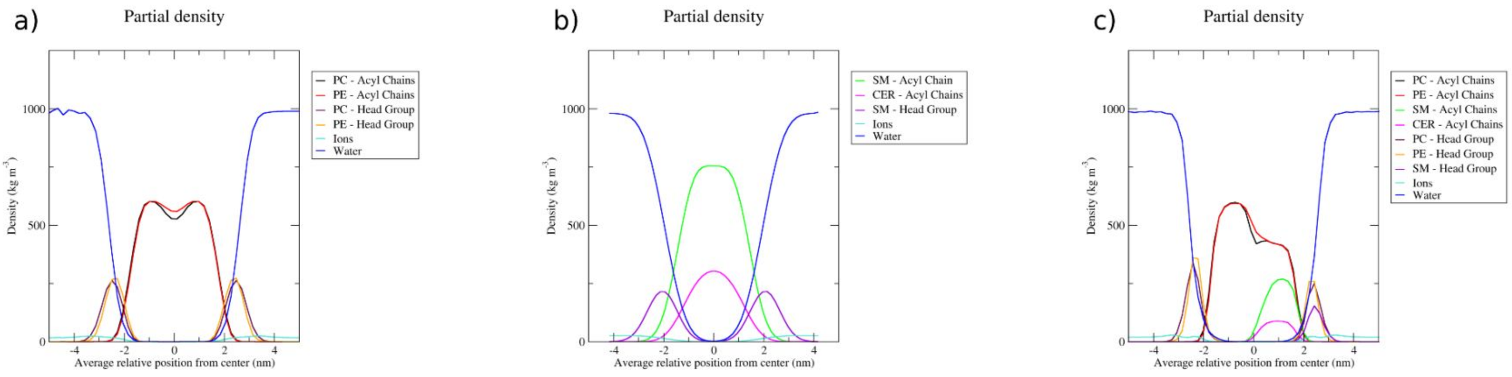

Figure SI-3: Partial density profiles calculated for simulated systems a) CG1, b) CG2, and c) CG3. For a full description of each system, please refer to Table 1.

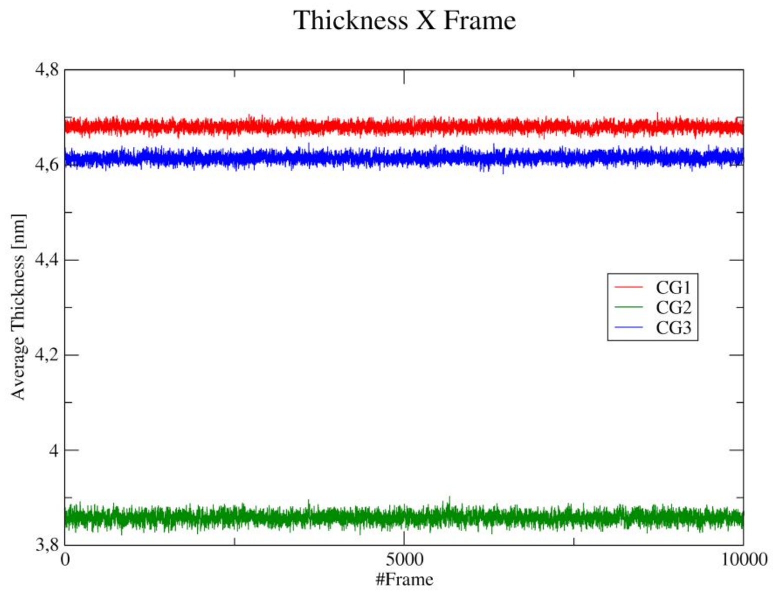

Figure SI-4: Average membrane thickness calculated for simulated systems a) CG1, b) CG2, and c) CG3. For a full description of each system, please refer to Table 1.

Computational Variables and Brief Definitions	
A_L	Molecular area or area per lipid is given by the ratio of the total surface area *A* for the number of lipids in each leaflet of a simulated membrane. (1,2)
S_c	The average membrane curvature or average curvature order parameter gives the distribution of the angle θ between the *z*-direction and the normal vector of the surface rectangular grid partition of the bilayer surface. When it is equal a value of 1, the partition vector is parallel to the vector normal to the surface system, what indicates a planar bilayer and deviations from this value means curved surfaces. (1,2)
Thickness	The average thickness is calculated between the two leaflets of the bilayer. For each rectangular grid partition of the surface the thickness, is calculated as the distance between the *z*-coordinate of both surfaces. (1,2)
Density Profile	The density profile refers to the distribution of mass as a function of its position thought time. The density profiles for selected chemical groups were calculated for the simulated systems using the grid fitting approaches. (1,2)
Lateral Pressure Profile	The lateral pressure profile describes how pressure is distributed within the plane of a system. It is calculated from the stress tensor, that includes normal stress, perpendicular to a surface, and shear stress, parallel to the surface. (3)

Table SI-1: Description of computational variables.

REFERENCES

(1) Santos, D. E. S.; Pontes, F. J. S.; Lins, R. D.; Coutinho, K.; Soares, T. A. SuAVE: A Tool for Analyzing Curvature-Dependent Properties in Chemical Interfaces. *J Chem Inf Model* **2020**, *60* (2), 473–484. DOI:10.1021/acs.jcim.9b00569.

(2) Santos, D. E. S.; Coutinho, K.; Soares, T. A. Surface Assessment via Grid Evaluation (SuAVE) for Every Surface Curvature and Cavity Shape. *J Chem Inf Model* **2022**, *62* (19), 4690–4701. DOI:10.1021/acs.jcim.2c00673.

(3) Vanegas, J. M.; Torres-Sánchez, A.; Arroyo, M. Importance of Force Decomposition for Local Stress Calculations in Biomembrane Molecular Simulations. *J Chem Theory Comput* **2014**, *10* (2), 691–702. DOI:10.1021/ct4008926.
